# Supplementary material for: Ultra-dense dislocations stabilized in high entropy oxide ceramics
Source: Nat Commun. 2022 May 24;13:2871. doi: 10.1038/s41467-022-30260-4 (PMC9130511; doi:10.1038/s41467-022-30260-4)
Supplement: Supplementary file 1 — Supplementary Information [file 41467_2022_30260_MOESM1_ESM.pdf]

Supplementary Materials for Ultra-dense dislocations stabilized in high entropy oxide ceramics

Yi Han[1#], Xiangyang Liu[1#], Qiqi Zhang[2], Muzhang Huang[1], Yi Li[1], Wei Pan[1*], Peng-an Zong[1], Lieyang Li[1], Zesheng Yang[1], Yingjie Feng[1], Peng Zhang[1, 3*], Chunlei Wan[1*]

[1] *State Key Laboratory of New Ceramics and Fine Processing, School of Materials Science and Engineering, Tsinghua University, Beijing 100084, China*

[2] *National Center for Electron Microscopy in Beijing, Beijing 100084, China*

[3] *Institute of Welding and Surface Engineering Technology, Faculty of Materials and Manufacturing, 100 Pingleyuan, Chaoyang District, Beijing 100124, China*

[#]These authors contributed equally: Yi Han, Xiangyang Liu.

[*]These authors jointly supervised this work: Wei Pan (email: panw@mail.tsinghua.edu.cn), Peng Zhang (email: peng.zhang.11@hotmail.com), Chunlei Wan (email: wancl@mail.tsinghua.edu.cn).

PDF file includes:

Supplementary Notes
Supplementary Figures 1 to 20
Supplementary Table 1

Other Supplementary Materials for this manuscript include the following:

Supplementary movie 1: Crack propagation behavior in $Gd_2Zr_2O_7$ model.
Supplementary movie 2: Crack bridging in HEPO during propagation process.
Supplementary movie 3: Crack deflection in HEPO during propagation process.

Contents

1. Modelling creation (Modelling and calculating details)........ 1
2. Classic ionic potential........ 1
3. HAADF-STEM imaging of HEFO........ 3
4. Calculation of dislocation density........ 3
5. The atomic energy near the dislocation core........ 12
6. Grain size distribution........ 14
7. Surface crack propagation path in HEPO........ 15
8. Fracture toughness of HEFO........ 16

Supplementary References........ 17

SUPPLEMENTARY NOTES

1. Modelling creation (Modelling and calculating details)

The rigid-ion mode with two opposite <111> {10-1} edge dislocations was created by a conjugate gradient (CG) structure optimization, which were successfully used to construct edge dislocation in ceramics recently[1, 2]. In the first step, a cubic zirconia model with a size of 28.850×106.387×199.879 Å^3 were constructed by ATOMSK software[3], in which x-axis, y-axis and z-axis was along the <10-1>, <111> and <1-21> direction, respectively. A vacuum slab was subsequently introduced by removing a single {111} lattice plane from $z = 1/4L_z$ to $z = 3/4L_z$ ($y = 1/2L_y$). The rare-earth zirconate model and high-entropy zirconate model were built by substituting Zr^{4+} ions with designed amount of RE^{3+} ions (RE = Sm, Gd, Dy, Er, Yb). To maintain the charge neutrality, 12.5 mol% oxygen vacancy was then introduced in this model by randomly removing O^{2-} ions. In the last step, the CG structure optimization process was performed to insert two symmetric edge dislocations with opposite Burgers vectors in the corresponding model (49962 atoms). The energy distribution is calculated based on the above model. For strain energy calculation, a more efficient model with a size of 107.354×30.991×57.141 Å^3 is used for all of the zirconates (including $Sm_2Zr_2O_7$, $Gd_2Zr_2O_7$, $Dy_2Zr_2O_7$, $Er_2Zr_2O_7$, $Yb_2Zr_2O_7$, $(Gd_{0.5}Er_{0.5})_2Zr_2O_7$, $(Sm_{1/3}Dy_{1/3}Yb_{1/3})_2Zr_2O_7$, $(Sm_{0.25}Gd_{0.25}Yb_{0.25}Er_{0.25})_2Zr_2O_7$, and $(Sm_{0.25}Gd_{0.25}Dy_{0.25}Yb_{0.25}Er_{0.25})_2Zr_2O_7$).

For high-entropy pyrochlore oxides (HEPO), a model with a size of 30.5272×105.749×225.055 Å^3 was created to calculated the energy distribution of the edge dislocation core. The same optimal process was applied by relaxing the HEPO model at 1873 K for 1ns before calculating the energy distribution.

A model with a size of 107.354×30.991×57.141 Å^3 was created to analyze the interaction between crack and dislocation in high-entropy fluorite oxides (HEFO) model. The Burge vector is 1/2[111] in the HEFO model. Two models with size of 199.879×28.85×100.387 Å^3 and 88.669×41.799×177.611 Å^3 were created to analyze the interaction between crack and dislocation in the HEPO samples. The Burge vectors are 1/2[111] and 1/2 [110], respectively.

All the figures of models with different specifications were created via OVITO software[4].

2. Classic ionic potential

Classic molecular dynamic (MD) simulations were performed by using LAMMPS code[5]. The inter-ionic potential, including a short-range Born-Maryer-Buckingham (BMB) and a long-range Coulombic potential (Equation (1)), was used to describe the interaction between different ions, which were successfully used to predict the phase transition of yttria stabilized zirconia and stability

of pyrochlore structure rare earth zirconate[6].

$$V(r_{ij}) = A_{ij}e^{-\frac{r_{ij}}{\rho_{ij}}} - \frac{C_{ij}}{r_{ij}^6} + \frac{1}{4\pi\varepsilon_0}\frac{q_i q_j}{r_{ij}} \quad (1)$$

In Equation (1), A_{ij}, C_{ij} and ρ_{ij} are interaction parameters, r_{ij} is the distance between ion i and j, q_i and q_j are the ionic charge of ion i and j, respectively. The first item in Equation (1) captured the repulsive force due to the overlapping of electron orbitals, the second and third items represented the attractive force from dispersion interaction and Coulombic forces. The potential parameters were listed in Supplementary Table 1.

The strain energy was estimated by calculating the energy difference between the dislocation model (with edge dislocations) and perfect model (without any dislocations). It could be regarded as the energy barrier for an edge dislocation at <111> direction at 1273 K.

$$E_{strain} = \frac{E_{edge} - E_{perf}}{2l} \quad (2)$$

where l is the length of the dislocation line, E_{strian}, E_{edge}, and E_{perf} are the strain energy, energy of the dislocation model and perfect model, respectively. Before calculating the configuration energy, both models were relaxed at 1873 K for 1 ns to eliminate the internal stress. After that, the average potential energy of last 10 configurations were calculated as the energy of models. The mixed law was also used to estimate the edge strain energy of edge dislocations in high-entropy (HE) model, i.e., the average strain energy of five mono rare-earth zirconates, including $Sm_2Zr_2O_7$, $Gd_2Zr_2O_7$, $Dy_2Zr_2O_7$, $Er_2Zr_2O_7$ and $Yb_2Zr_2O_7$.

Supplementary Table 1 Potential parameters applied in molecular dynamic simulation[6, 7, 8, 9].

Species	A_{ij} (eV)	ρ_{ij} (Å)	C_{ij} (eV Å^6)
O^{2-}- O^{2-}	9547.96	0.2192	32
Zr^{4+}- O^{2-}	1502.11	0.3477	5.1
Sm^{3+}- O^{2-}	1944.44	0.3414	21.49
Gd^{3+}- O^{2-}	1885.75	0.3399	20.34
Dy^{3+}- O^{2-}	1807.84	0.3393	18.77
Er^{3+}- O^{2-}	1739.91	0.3389	17.55
Yb^{3+}- O^{2-}	1649.80	0.3386	16.57
Ta^{5+}-O^{2-}	1315.57	0.36905	0
Nb^{5+}-O^{2-}	985.869	0.376	0
Ca^{2+}-O^{2-}	1227.7	0.3372	0

3. HAADF-STEM imaging of HEFO

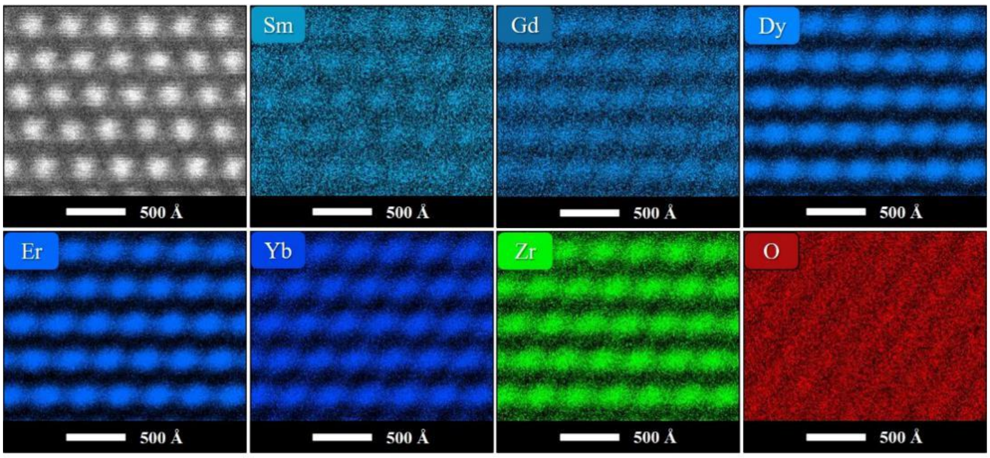

Supplementary Figure 1 | Atomic resolution HAADF-STEM imaging of HEFO sample. All elements are homogeneously distributed without composition segregation.

4. Calculation of dislocation density

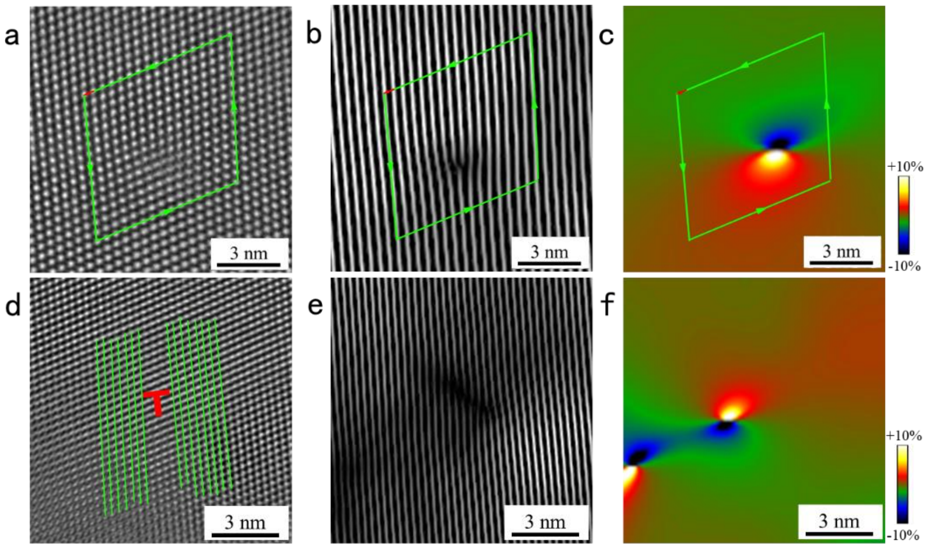

Supplementary Figure 2 | Locating dislocations in HRTEM images. (a) Dislocation with Burgers circuit; (b) FFT filtered image of (a); (c) GPA image of (a); (d) Locating with auxiliary lines; (e) FFT filtered image of (d); (f) GPA image of (d). The color scale represents change in strain intensity from −10% (compressive) to 10% (tensile).

Dislocation densities were calculated through high-resolution transmission electron microscope (HRTEM) images. The inverse fast Fourier transform (IFFT) and geometric phase analysis (GPA) were used to assist the HRTEM image to fix the position of edge dislocations. The existence of edge dislocation was verified by drawing a Burgers circuit. As the image quality is significantly affected by the stress concentration around dislocations, auxiliary lines paralleling to the lattice plane were used to find dislocation (see Supplementary Figure 2(d)). To calculate the dislocation density, we

count the number of dislocations in three independent TEM images for all high-entropy oxide ceramics. The TEM image of ceramics with different structures are shown in Supplementary Figure 2 to 11.

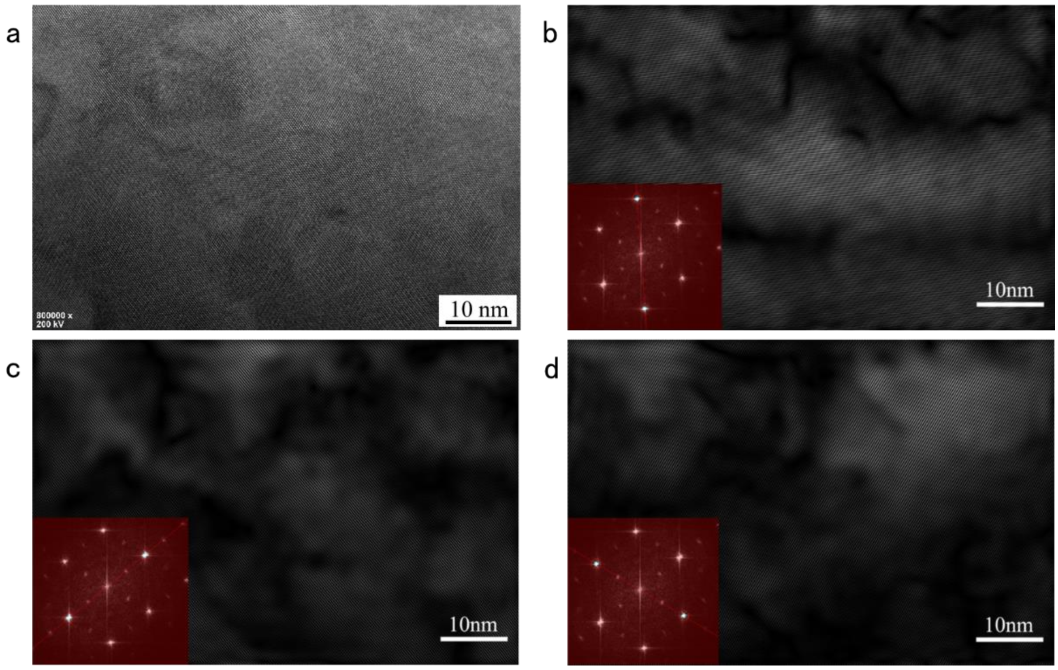

Supplementary Figure 3 | HRTEM observation, fast Fourier transform (FFT) filtered image of $Gd_2Zr_2O_7$. (a) HRTEM graph; (b-d) The FFT and inverse fast Fourier transform (IFFT) filtered image with different frequency regions in reciprocal space.

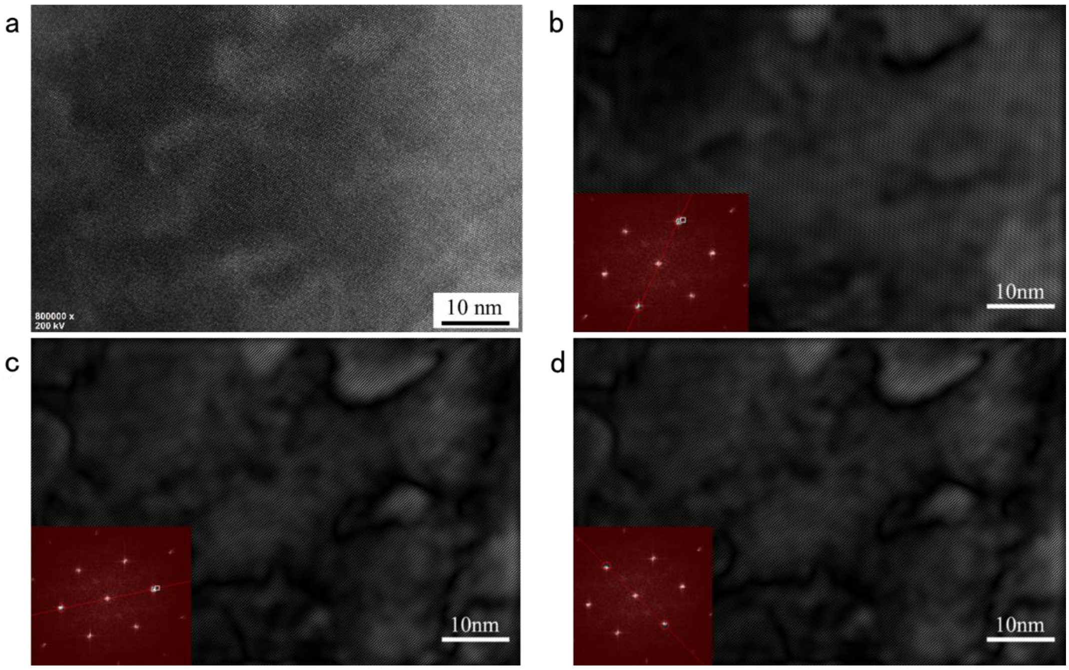

Supplementary Figure 4 | HRTEM observation, fast Fourier transform (FFT) filtered image of $(Gd_{0.5}Er_{0.5})_2Zr_2O_7$. (a) HRTEM graph; (b-d) The FFT and inverse fast Fourier transform (IFFT) filtered image with different frequency regions in reciprocal space.

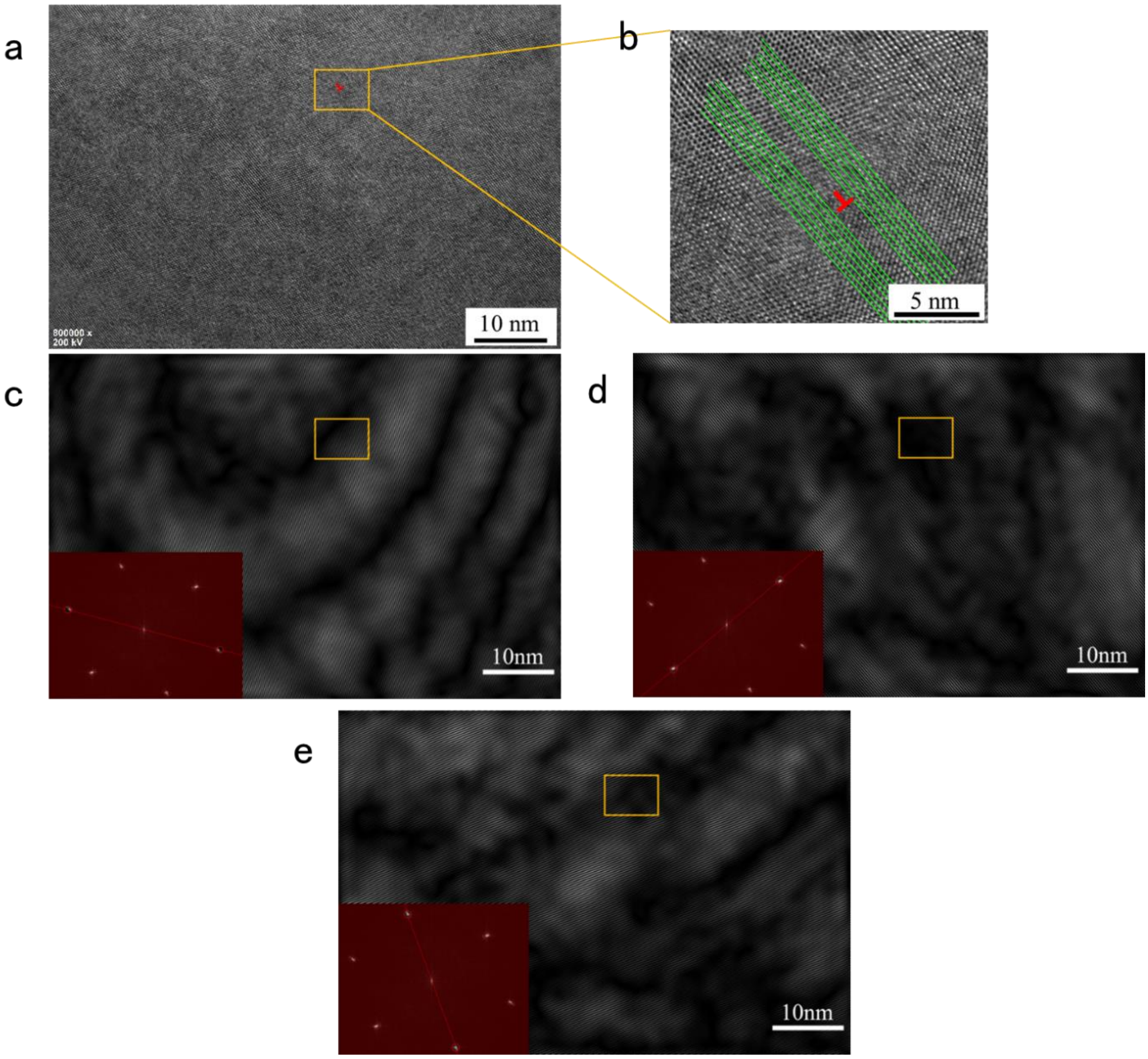

Supplementary Figure 5 | HRTEM observation, fast Fourier transform (FFT) filtered image of $(Sm_{1/3}Dy_{1/3}Yb_{1/3})_2Zr_2O_7$. (a) HRTEM graph; (b) HRTEM graph of single edge dislocation; (c-e) The FFT and inverse fast Fourier transform (IFFT) filtered image with different frequency regions in reciprocal space. The dislocations are marked by yellow squares.

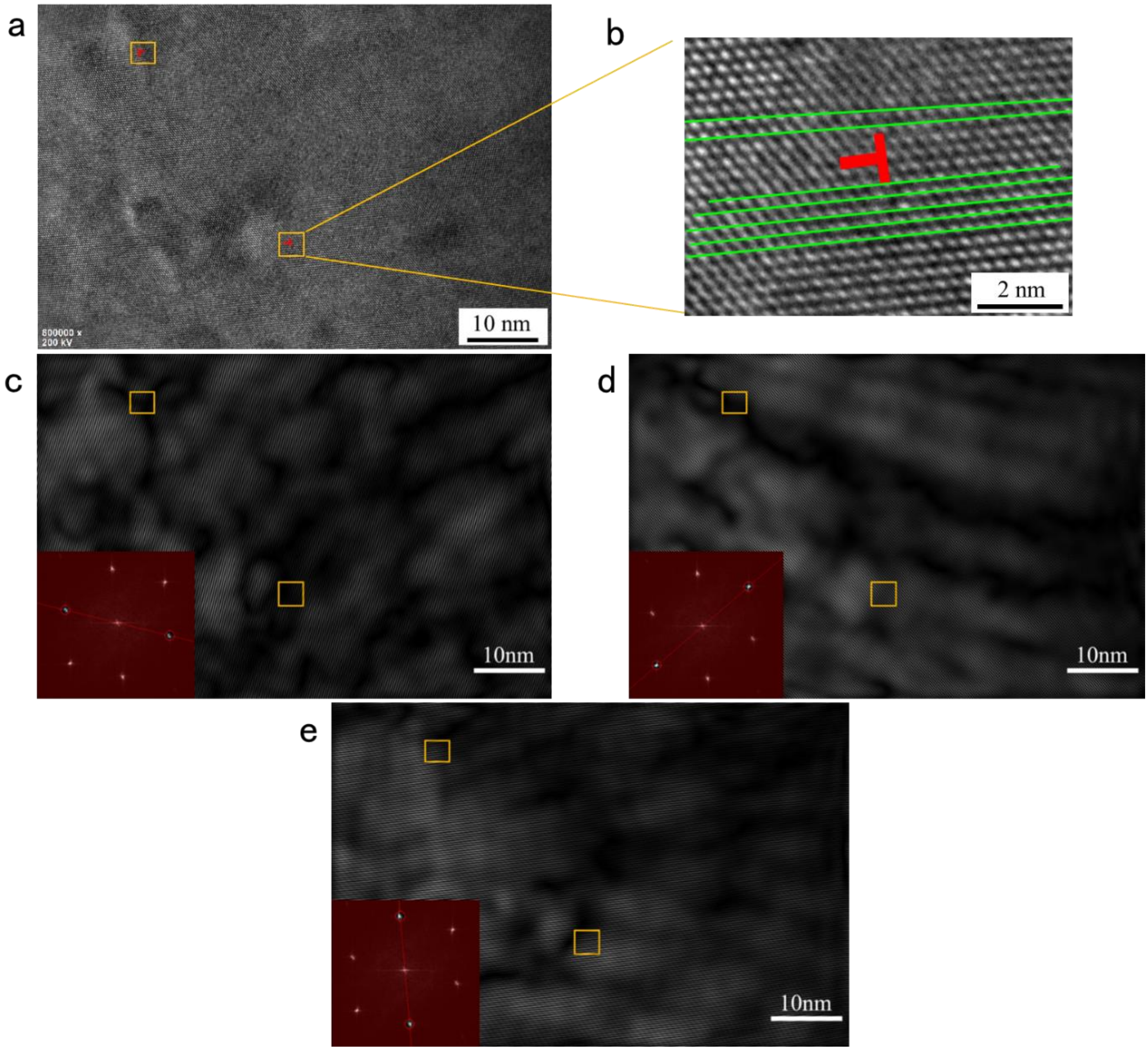

Supplementary Figure 6 | HRTEM observation, fast Fourier transform (FFT) filtered image of $(Sm_{0.25}Gd_{0.25}Er_{0.25}Yb_{0.25})_2Zr_2O_7$. (a) HRTEM graph; (b) HRTEM graph of single edge dislocation; (c-e) The FFT and inverse fast Fourier transform (IFFT) filtered image with different frequency regions in reciprocal space. The dislocations are marked by yellow squares.

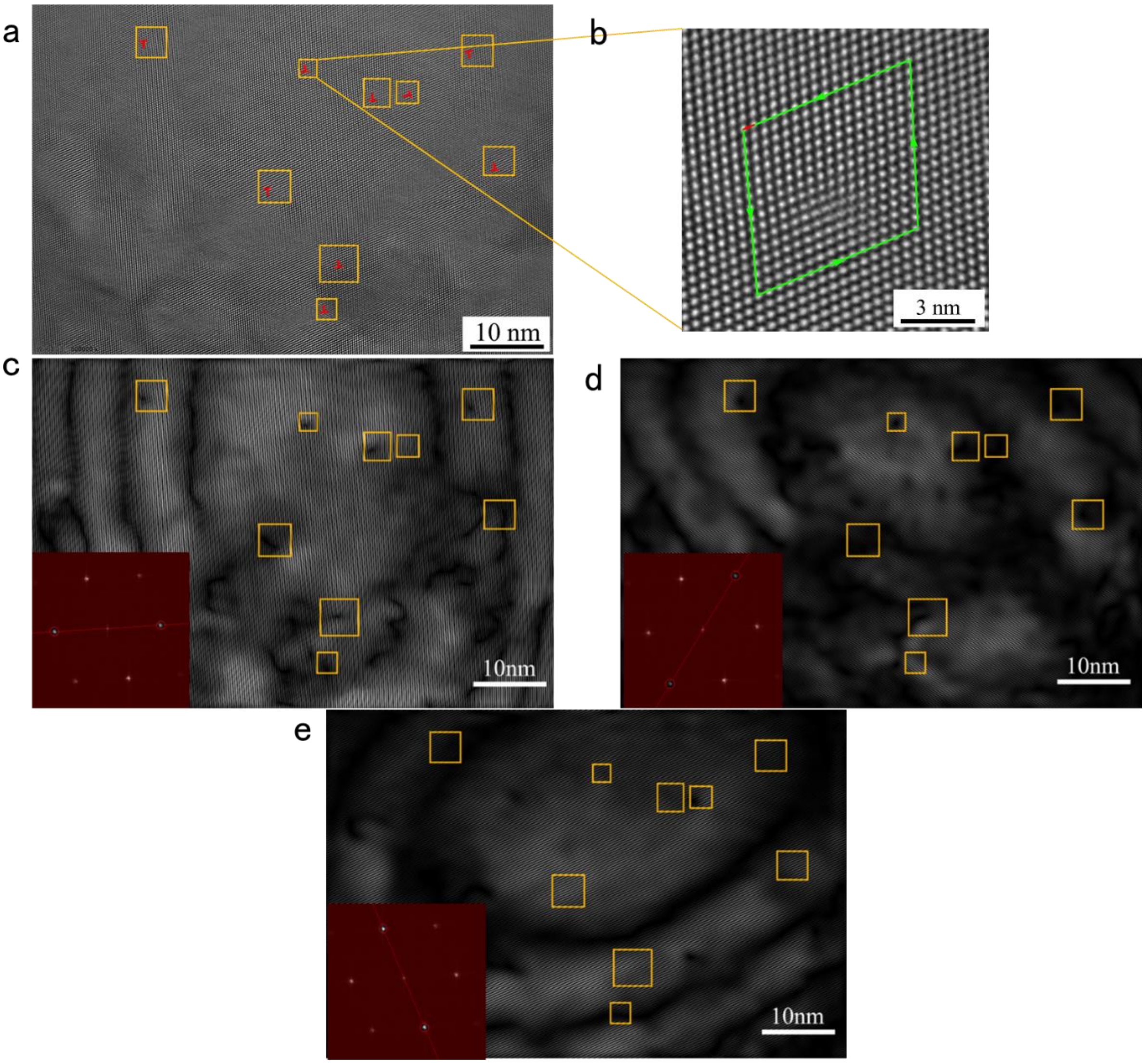

Supplementary Figure 7 | HRTEM observation, fast Fourier transform (FFT) filtered image of $(Sm_{0.2}Gd_{0.2}Dy_{0.2}Er_{0.2}Yb_{0.2})_2Zr_2O_7$. (a) HRTEM graph; (b) HRTEM graph of single edge dislocation; (c-e) The FFT and inverse fast Fourier transform (IFFT) filtered image with different frequency regions in reciprocal space. The dislocations are marked by yellow squares.

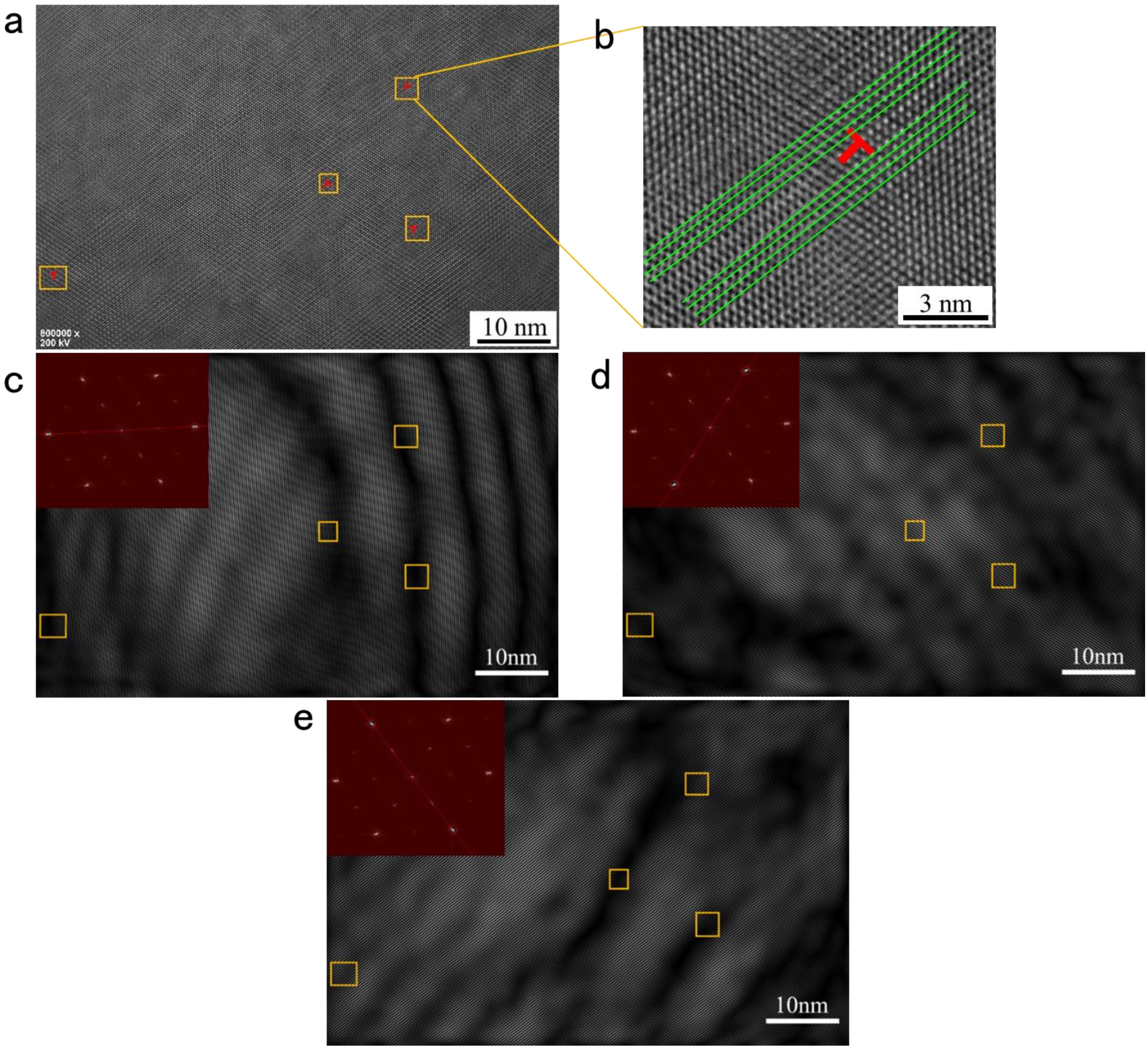

Supplementary Figure 8 | HRTEM observation, fast Fourier transform (FFT) filtered image of $Ca_{1.2}Gd_{0.8}Zr_{0.8}Nb_{0.6}Ta_{0.6}O_7$. (a) HRTEM graph; (b) HRTEM graph of single edge dislocation; (c-e) The FFT and inverse fast Fourier transform (IFFT) filtered image with different frequency regions in reciprocal space. The dislocations are marked by yellow squares.

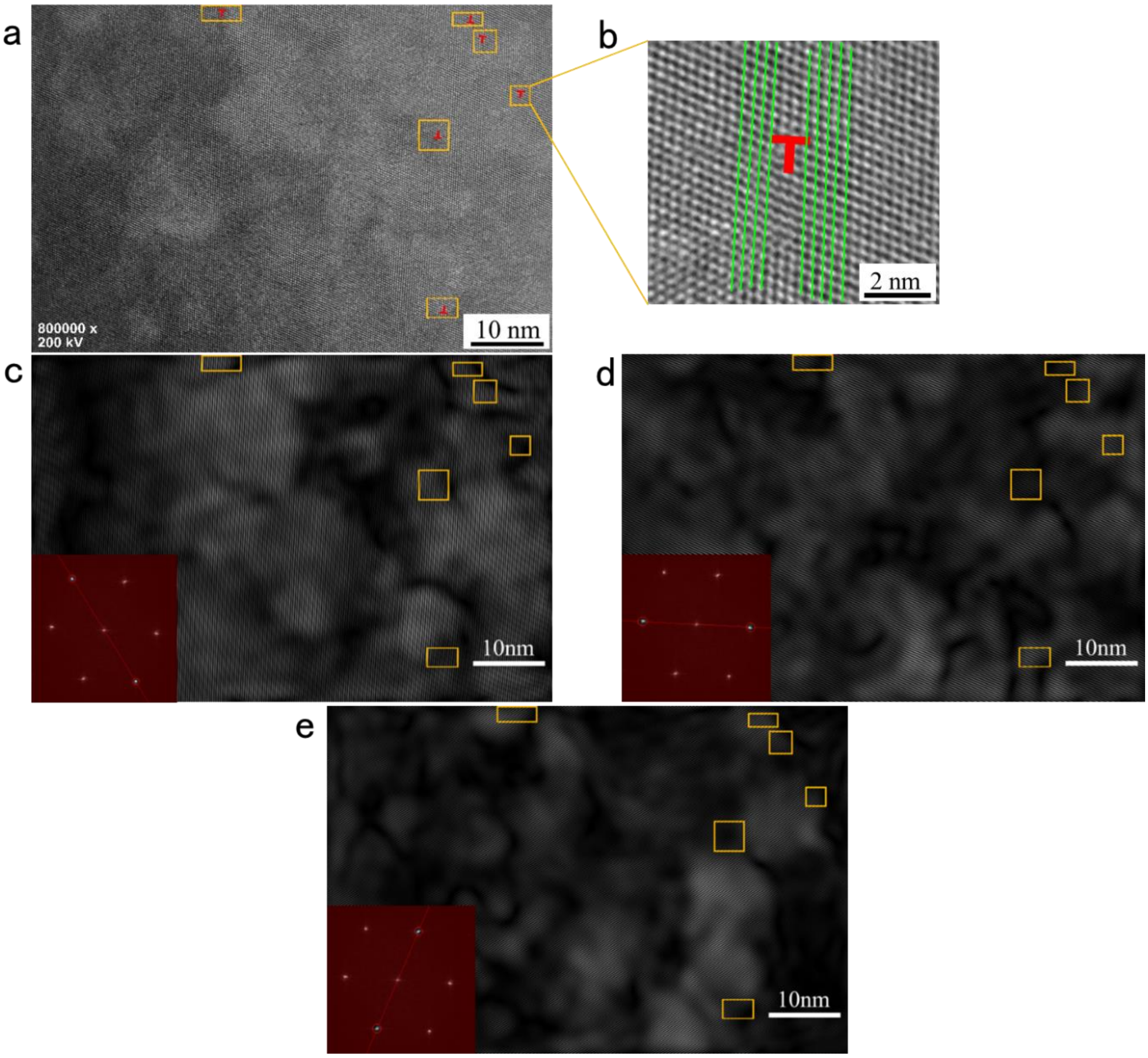

Supplementary Figure 9 | HRTEM observation, fast Fourier transform (FFT) filtered image of $(Dy_{0.25}Er_{0.25}Y_{0.25}Yb_{0.25})_3NbO_7$. (a) HRTEM graph; (b) HRTEM graph of single edge dislocation; (c-e) The FFT and inverse fast Fourier transform (IFFT) filtered image with different frequency regions in reciprocal space. The dislocations are marked by yellow squares.

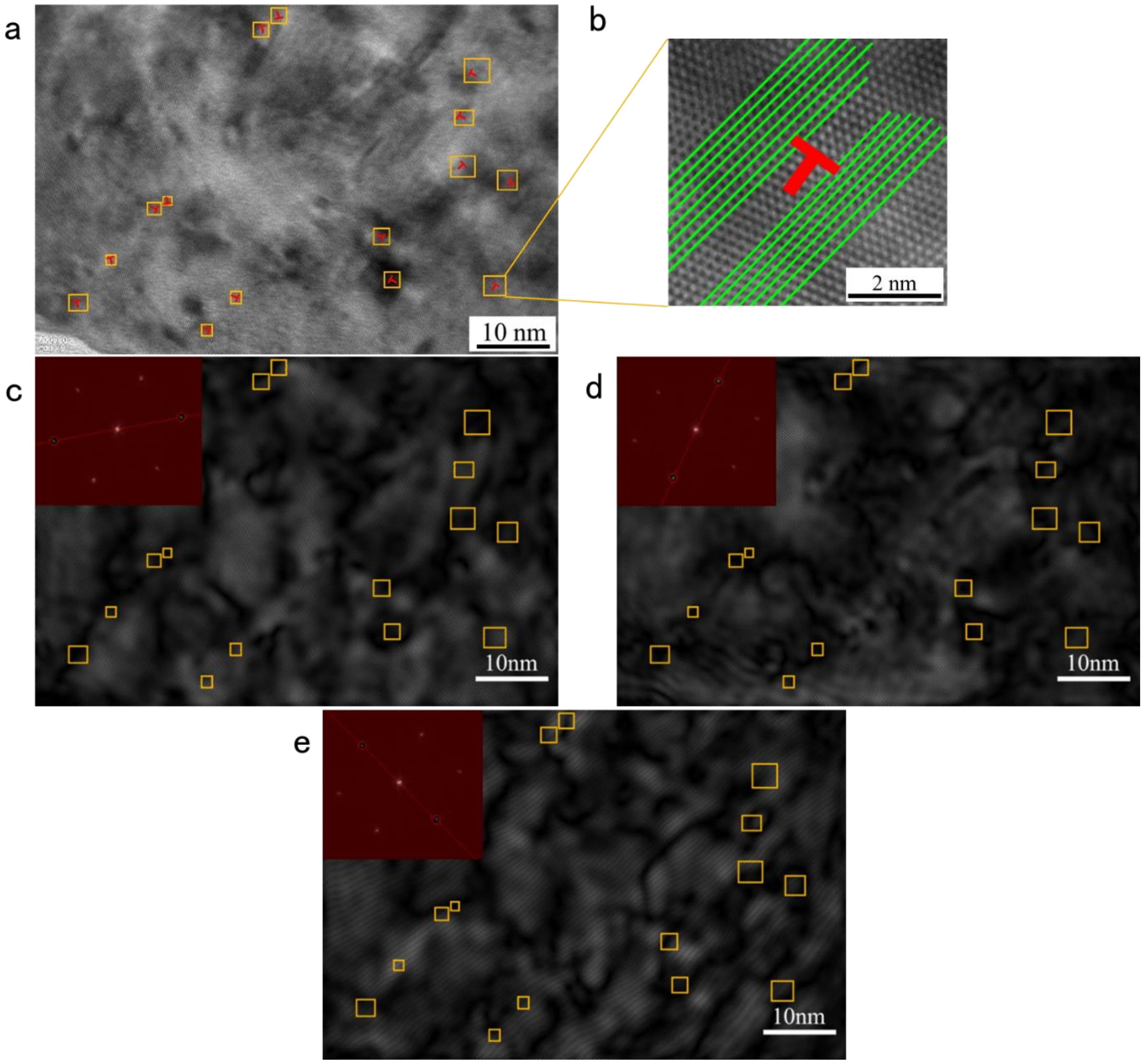

Supplementary Figure 10 | HRTEM observation, fast Fourier transform (FFT) filtered image of $Sr(Ti_{0.2}Y_{0.2}Zr_{0.2}Nb_{0.2}Sn_{0.2})O_3$. (a) HRTEM graph; (b) HRTEM graph of single edge dislocation; (c-e) The FFT and inverse fast Fourier transform (IFFT) filtered image with different frequency regions in reciprocal space. The dislocations are marked by yellow squares.

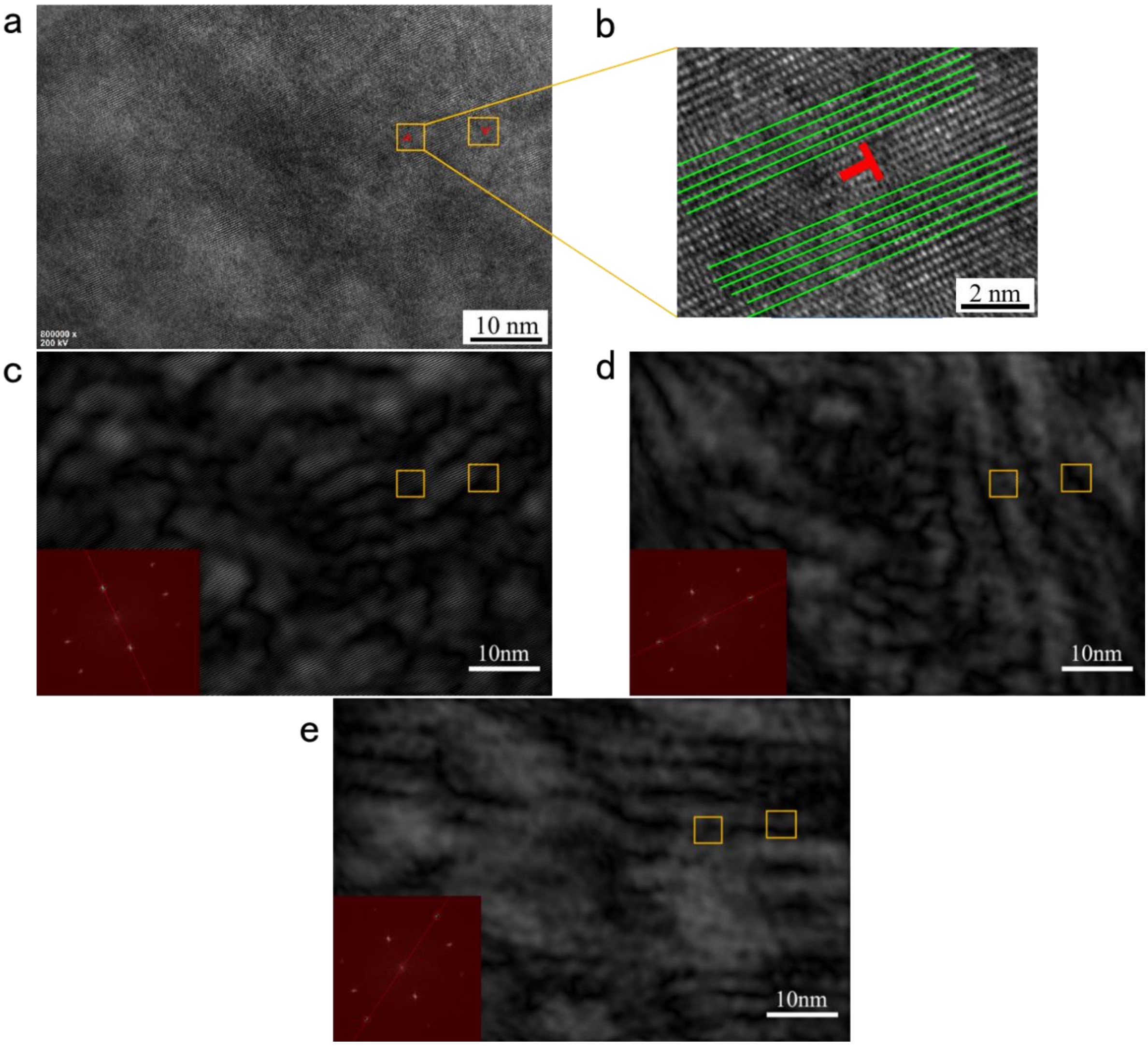

Supplementary Figure 11 | HRTEM observation, fast Fourier transform (FFT) filtered image of $(Nd_{0.2}Sm_{0.2}Gd_{0.2}Er_{0.2}Yb_{0.2})NbO_4$. (a) HRTEM graph; (b) HRTEM graph of single edge dislocation; (c-e) The FFT and inverse fast Fourier transform (IFFT) filtered image with different frequency regions in reciprocal space. The dislocations are marked by yellow squares.

5. The atomic energy near the dislocation core

The energy distribution of rare-earth cations in the HEFO model are shown in SupplementarySupplementary Figure **12**. Then the maximum range that a dislocation core effected can be defined at the starting point of the level lines (cut off radii) in Supplementary Figure 12, which is about 2 nm.

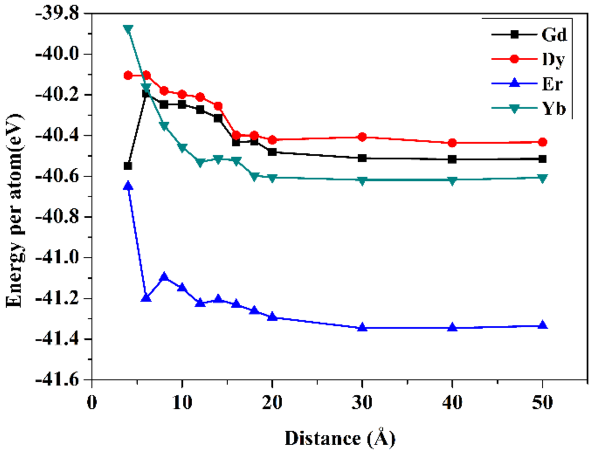

Supplementary Figure 12 | The atomic energy of each species as a function of distance to dislocation core.

The energy distribution of edge dislocation core in HEPO was calculated by MD simulation. The calculation details and model creation has been depicted in previous section. In order to maintain the charge neutrality, four edge dislocations were created by deleting two half (111)-lattice with a distance of 10 nm. Two stacking faults were also created in the model that did not observe in experimental results and corresponding references[10]. Therefore, the influence of stacking fault on the strain energy of edge dislocations in HEPO should be eliminated by subtracting the stacking fault energy. The initial model and optimized model were shown in Supplementary Figure 13. Clearly, the edge dislocations migrated to reduce the systematic energy. Strain was generated near the dislocation core and anti-phase boundary (APB) was also observed in the relaxed model shown in Supplementary Figure 14.

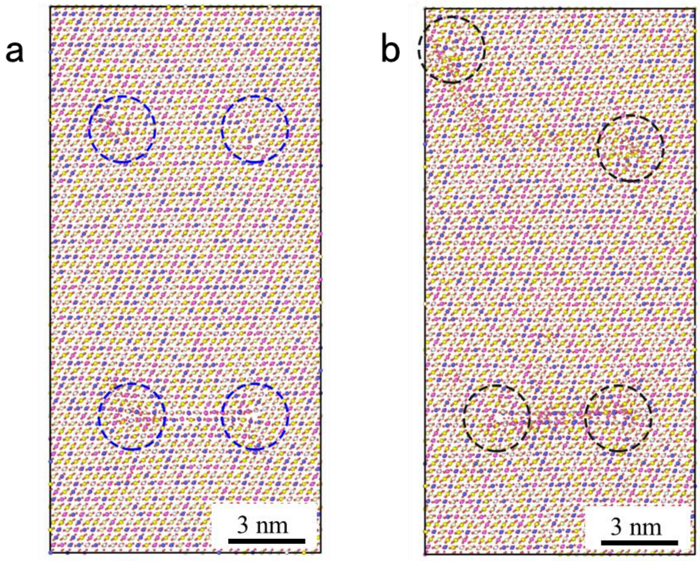

Figure 13 | The initial model and optimized model of HEPO. (a) The initial configuration of HEPO with four edge dislocations; (b) configuration of HEPO model optimized at 1873 K for 1 ns.

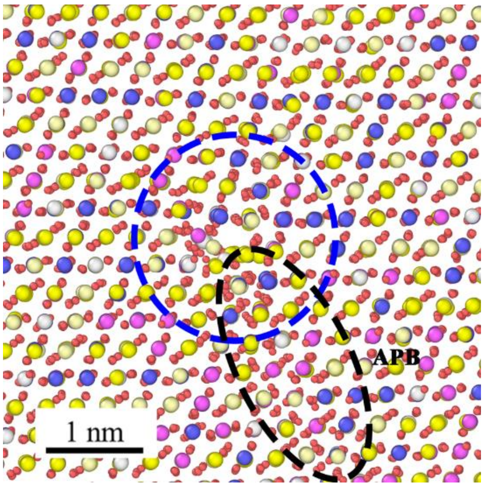

Supplementary Figure 14 | Configuration of dislocation core and anti-phase boundary in relaxed HEPO model.

The energy distribution of labeled core was statistically calculated in order to clarify the maximum influence range of the dislocation core in HEPO. The energy distribution of the core along with the distance was shown in Supplementary Figure 15. It can be seen, the cut off radii of the core lies between 3.3 nm and 4.0 nm, which is much larger than the cut off radii of the dislocation core in HEFO (2.0 nm). The larger cut off radii originating from the creation of APB boundary which are also verified by Gaboriaud RJ., Overview, 2020[10].

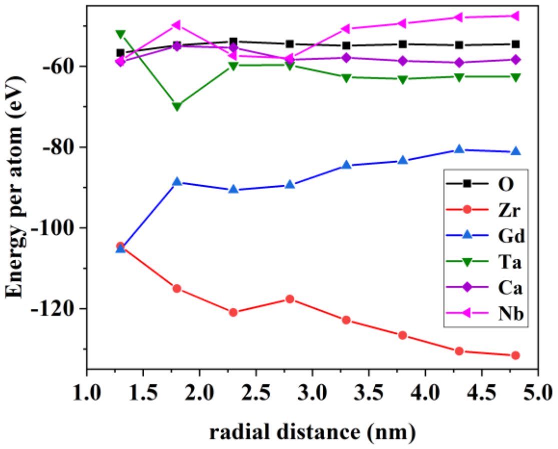

Supplementary Figure 15 | Distribution of the energy per atom along with the radial distance.

As a result, the toughening effect from edge dislocation in HEPO is more effective than the dislocations in HEFO, owing to the large strained region induced by dislocations. Though the dislocation density of HEFO is much higher than HEPO, the total toughening effect of dislocation in HEPO is still significantly higher than HEFO.

6. Grain size distribution

The SEM image of $Gd_2Zr_2O_7$, HEPO and HEFO with same magnification are shown in Supplementary Figure 16. Grain size distribution of $Gd_2Zr_2O_7$, HEPO and HEFO are analyzed by counting ~200 grains, which are shown in Supplementary Figure 17. The average grain size of $Gd_2Zr_2O_7$ is comparatively small with the value of 1.857 μm. The grain size distribution of HEFO is similar to the $Gd_2Zr_2O_7$ with the value of 2.071 μm. The average grain size of HEPO is little bit bigger than $Gd_2Zr_2O_7$ and HEPO with the average value of 2.215 μm.

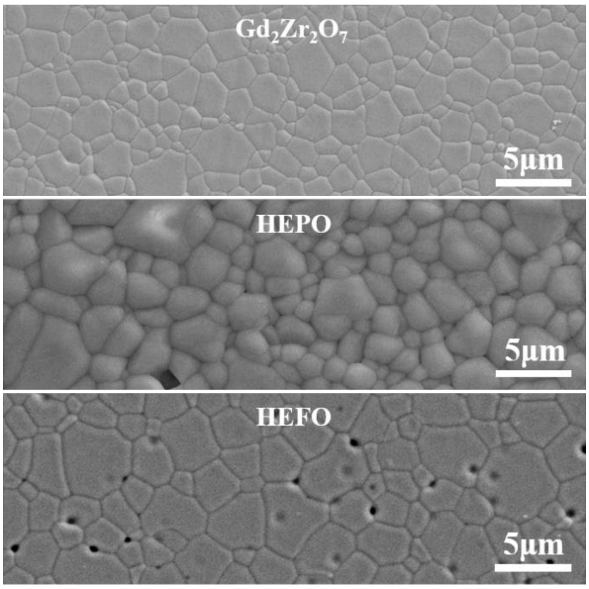

Supplementary Figure 16 | SEM image of $Gd_2Zr_2O_7$, HEPO and HEFO.

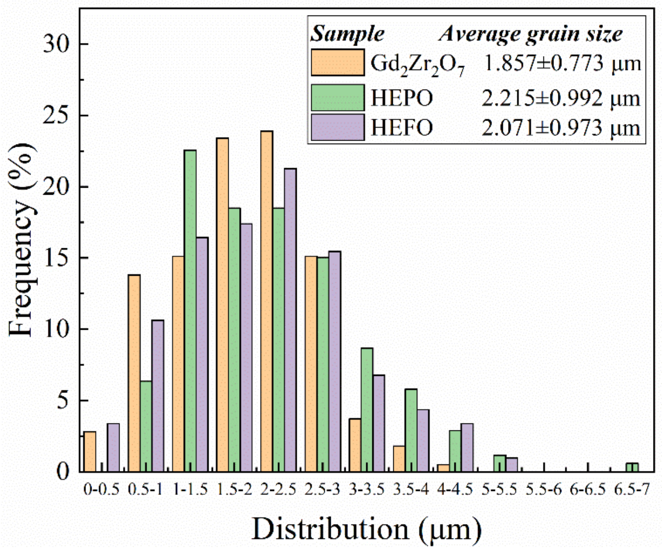

Supplementary Figure 17 | Grain size distribution of $Gd_2Zr_2O_7$, HEPO and HEFO.

7. Surface crack propagation path in HEPO

To give a more objective and comprehensive view on the crack propagation behavior in HEPO, more indentation tests were done to examine the crack propagation path. We have randomly selected eight independent cracks as shown in Supplementary Figure 18. It can be seen that most cracks show an intragranular cracking mode, in which the crack deflection and bridging are frequently observed and can be considered as the dominant toughening mechanism.

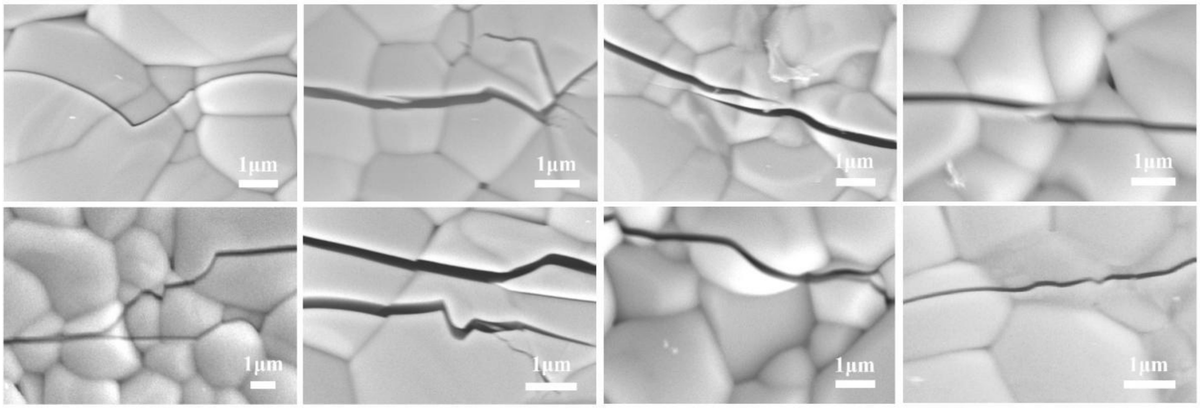

Supplementary Figure 18 | Intragranular deflection, bridging and divarication from different crack paths in HEPO simples.

8. Fracture toughness of HEFO

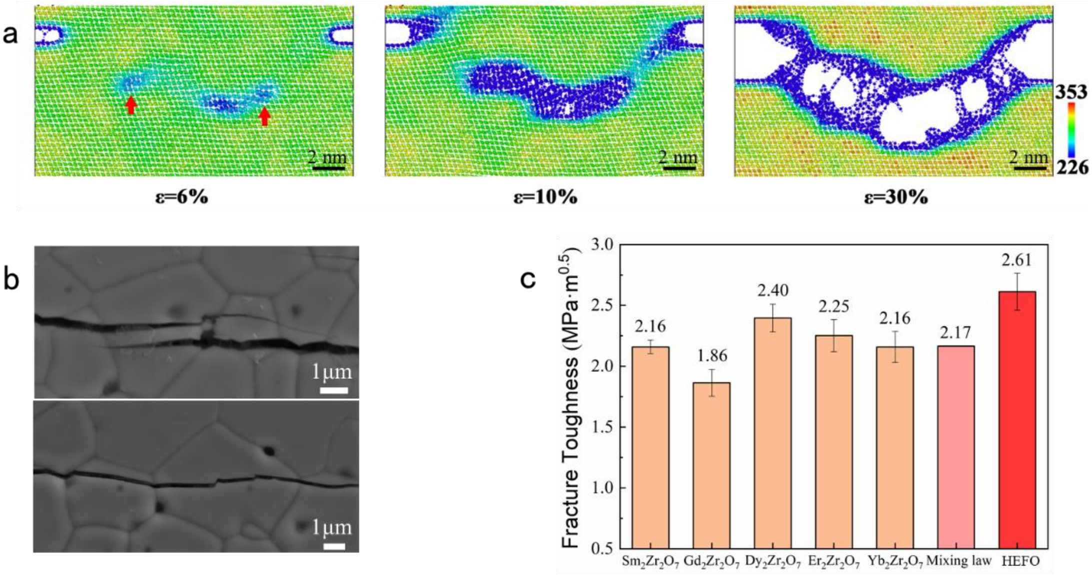

Supplementary Figure 19 | The crack propagation of $Sm_{0.4}Gd_{0.4}Dy_{0.4}Er_{0.4}Yb_{0.4}Zr_2O_7$ (HEFO) simulated by MD and experimental observations. (a) Crack deflection in HEFO interacting with two edge dislocations (dislocations are indicated by red arrows, and the color scale denotes the coordination number of each cation (unit: counts)); (b) The crack propagation path in HEFO sample; (c) fracture toughness of mono rare-earth zirconates, HEFO and the calculated value by mixing law. The error bars represent the standard deviation of calculated dislocation density.

Compared with the fracture toughness calculated by the mixing law, the fracture toughness of HEFO was improved approximately 20% shown in Supplementary Figure 19. The toughening effect is lower than HEPO that were explained in **Section 8**. In order to elucidate the difference of dislocations, the MD simulation was performed to compare the energy distribution near the dislocation core shown in Supplementary Figure 20. It is worth noting that we only observed the dislocations (core) in the atomic image, and did not observe the dislocation lines from the bright filed graph of the scanning transmission electron microscope (STEM). Therefore, it was supposed that the dislocation line was rather short that would only be a few atomic spacing. Moreover, the solid solution elements in HEPO had larger inhomogeneity of mass, valence, and electronegativity.

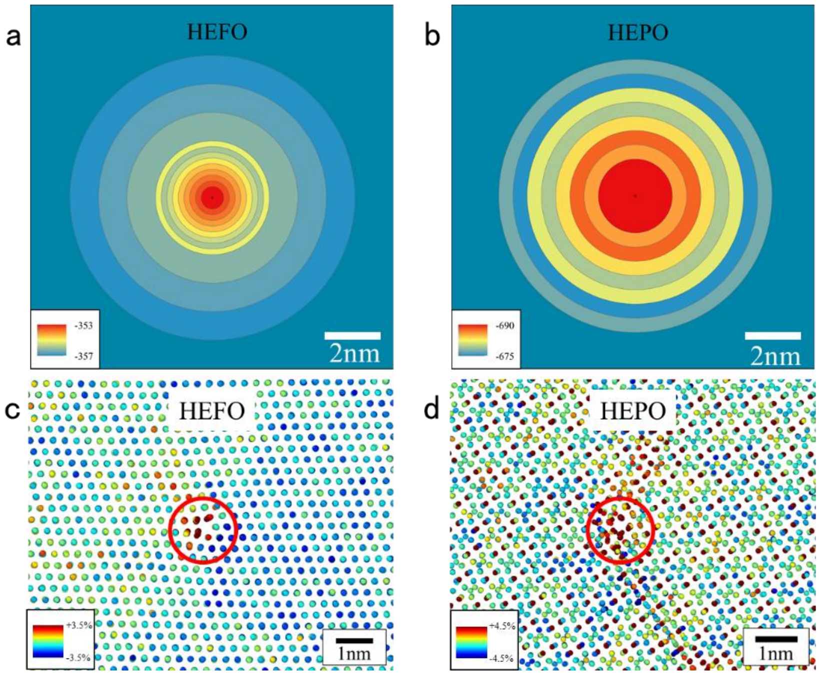

Supplementary Figure 20 | The difference of dislocations in HEFO and HEPO. (a) The energy distribution of HEFO along with the radial distance; (b) The energy distribution of HEPO (The color scale in (a) and (b) denotes the value of energy (eV)); (c) Voronoi atomic volumes distribution of the cations near the dislocation in HEFO; (d) Voronoi atomic volumes distribution of the anions near the dislocation in HEPO (dislocations are indicated by red circles). The color scale in (c) and (d) represents the value of strain (%).

Supplementary References

1. Sun L, Marrocchelli D, Yildiz B. Edge dislocation slows down oxide ion diffusion in doped CeO2 by segregation of charged defects. *Nat Commun* **6**, 6294 (2015).
2. Marrocchelli D, Sun L, Yildiz B. Dislocations in SrTiO3: Easy To Reduce but Not so Fast for Oxygen Transport. *J Am Chem Soc* **137**, 4735-4748 (2015).
3. Hirel P. Atomsk: A tool for manipulating and converting atomic data files. *Comput Phys Commun* **197**, 212-219 (2015).
4. Stukowski A. Visualization and analysis of atomistic simulation data with OVITo the Open Visualization Tool. *Modell Simul Mater Sci Eng* **18**, 015012 (2009).
5. Plimpton S. Fast Parallel Algorithms for Short-Range Molecular Dynamics. *J Comput Phys* **117**, 1-19 (1995).
6. Stanek CR, Minervini L, Grimes RW. Nonstoichiometry in A2B2O7 Pyrochlores. *J Am Ceram Soc* **85**, 2792-2798 (2002).
7. Lewis GV, Catlow CRA. Potential models for ionic oxides. *J Phys C: Solid State Phys* **18**, 1149-1161 (1985).
8. Yang J*, et al.* Diffused Lattice Vibration and Ultralow Thermal Conductivity in the Binary Ln–Nb–O Oxide System. *Adv Mater* **31**, 1808222 (2019).
9. Pirovano C, Islam MS, Vannier R-N, Nowogrocki G, Mairesse G. Modelling the crystal structures of Aurivillius phases. *Solid State Ionics* **140**, 115-123 (2001).
10. Gaboriaud RJ. *Dislocations in Anion-Deficient Fluorite-type Oxides under harsh environment: RE2O3, Pyrochlores,*

δ-Phase. Overview (2020).
