# Supplementary material for: Ultra-dense dislocations stabilized in high entropy oxide ceramics
Source: Nat Commun. 2022 May 24;13:2871. doi: 10.1038/s41467-022-30260-4 (PMC9130511; doi:10.1038/s41467-022-30260-4)
Supplement: Supplementary file 3 — Description of Additional Supplementary Files [file 41467_2022_30260_MOESM3_ESM.pdf]

Description of Additional Supplementary Files

File Name: Supplementary Movie 1
Description: Crack propagation behavior in the $Gd_2Zr_2O_7$ model.

File Name: Supplementary Movie 2
Description: Crack bridging in HEPO during propagation process.

File Name: Supplementary Movie 3
Description: Crack deflection in HEPO during propagation process.
